# Effect of short video addiction on the sleep quality of college students: chain intermediary effects of physical activity and procrastination behavior

**DOI:** 10.3389/fpsyg.2023.1287735

**Published:** 2024-01-11

**Authors:** Zhe Zhao, Yali Kou

**Affiliations:** ^1^Department of Physical Education, Kunsan National University, Gunsan, Republic of Korea; ^2^School of Marxism, Shangqiu Normal University, Shangqiu, Henan, China

**Keywords:** short video addiction, sleep quality, physical activity, procrastination behavior, college students

## Abstract

**Introduction:**

The purpose of this study was to examine the impact of short video addiction on college students' sleep quality and to elucidate the mechanism underlying this relationship. Thus, we examined the correlation between short video addiction and sleep quality and analyzed the roles of physical activity and procrastination.

**Methods:**

The Short Video Addiction Scale, Pittsburgh Sleep Quality Index, Aitken Procrastination Inventory, and Physical Activity Rating Scale were administered to 337 college students. Data were analyzed using SPSS 27.0. Pearson's correlation analysis and mediation analysis using the bootstrapping test were performed for the standard method bias test.

**Results:**

(1) Overall, 25.2% of college students had problems with sleep quality (indicated by a PSQI score ≥ 8). (2) Short video addiction score is positively correlated with college students' sleep quality score; procrastination score was positively associated with both short video addiction score and sleep quality score, and physical activity score was negatively associated with them. (3) Short video addiction significantly positive predicted sleep quality (β = 0.458, *P* < 0.001), a significant negative predictive effect on physical exercise (β = −0.183, *P* < 0.001), and a significant positive effect on procrastination behavior (β = 0.246, *P* < 0.001). After physical exercise and procrastination behavior were entered into the regression equation, short video addiction and procrastination were significantly positive predictors of sleep quality, and physical activity was significantly negative predictor of sleep quality. (4) After accounting for the variables of age, gender, and grade, physical activity and procrastination behaviors independently mediated the association between short-video addiction and sleep quality. Physical activity and procrastination behavior acted as chain mediators in the association between short video addiction and sleep quality, with a chain mediation effect percentage of 1.04%. Short video addiction directly affects college students' sleep quality, indirectly through physical activity and procrastination behavior.

## Introduction

Since short video platforms are growing so rapidly, short video applications are beginning to be fully integrated into the lives of college students. In China, the proportion of Internet users who engage with short videos is at 94% (China Internet Network Information Center, 2023). The main users of short video apps are college students; as of June 2022, a survey found that college students accounted for 47.7% of the 6,171 young users aged 18–23 years old who used the short video app TikTok (Mega Arithmetic, 2022). The ease of use and entertaining features of short video apps reinforce the tendency of college students to overuse them, making it challenging to suppress impulses. Despite experiencing negative consequences, young people still spend considerable time using these short video apps (Rathakrishnan et al., 2021). Short video apps enrich college student's lives, but short video addiction due to overuse is increasing (Pu et al., 2023). Short video addiction is a state of obsession in which individuals lose control of their short video behaviors by using them repetitively, thereby creating a persistent and strong sense of need and dependence that seriously affects their study and life and negatively impacts their mental health (Qin et al., 2019). This behavioral addiction can evolve from smartphone addiction. Several research studies have indicated a correlation between smartphone addiction and sleep quality, whereby an excessive reliance on smartphones is found to be connected with suboptimal sleep quality among college students (Zhou et al., 2019; Xie et al., 2020; Guo et al., 2023). Insufficient scholarly investigation exists regarding the inherent processes and moderating functions of short-video addiction in relation to the sleep quality. Studying the effects of short video addiction on college students' sleep quality not only expands the scope of research on factors affecting sleep quality at the theoretical level but also this study offers tangible scientific information that can assist college students in enhancing their sleep quality, specifically from the standpoint of media psychology.

### Short video addiction and sleep quality

According to the uses and gratifications theory the satisfaction related to an individual's need to use a medium will motivate his or her continued use (Katz and Blumler, 1976), and short videos influence addiction by activating the user's perceived enjoyment (Tian et al., 2023). The use of smartphones, especially during sleep, is the most vital factor associated with sleep disruption (Aili et al., 2017; Dissing et al., 2022). It has been demonstrated that routine TikTok use is linked to cognitive arousal before bed, which causes poor sleep (Wang and Scherr, 2022). The use of various short video applications is one of the major causes of reduced sleep quality in the college population. Accordingly, this study proposed Hypothesis 1: Short video addiction among college students significantly predicts sleep quality.

### The mediating role of physical activity

Physical activity is defined as an activity that increases energy in the body at the level of basal metabolism and refers to physical activity produced by the contraction of skeletal muscles. Studies have shown an important link between physical activity and sleep (Ghrouz et al., 2019). Physical activity can help individuals relieve stress, improving sleep quality (Zhai et al., 2021). A survey of a group of college students in China over three consecutive years found an association between physical activity and sleep (Semplonius and Willoughby, 2018). In addition, moderate physical activity facilitates sleep quality in young people (Wang and Boros, 2021). Studies of college students from different countries have found that most students with poor sleep quality lack physical activity (Mahfouz et al., 2020). Moreover, regular physical activity is necessary to improve sleep quality (Zhao et al., 2023). Physically inactive teenage students more inclined to have smartphone use problems (Pereira et al., 2020). Physical activity indirectly improves sleep quality through the bedtime delay of the short video application TikTok (Zhang X. et al., 2022). Based on the above studies on physical activity, short video addiction, and sleep quality, proposed Hypothesis 2: Physical activity mediates the relationship between short video addiction and sleep quality.

### The mediating role of procrastination behavior

Procrastination behavior has been one of the major influencing factors that causes difficulties in college students' studies and lives. Procrastination is a behavior in which an individual voluntarily postpones action on a task that must be completed despite anticipating the negative consequences of the behavior (Tian, 2019). Previous studies have pointed out that procrastination leads to decreased sleep quality and is a positive predictor of sleep problems (Fuschia et al., 2015; Ma et al., 2022). In contrast, sleep problems negatively predict procrastination (Aneta et al., 2019). Research shows that bedtime procrastination severely impacts the timing and duration of a teen's sleep (Pu et al., 2022), and reducing bedtime procrastination can help college students improve their sleep quality, especially for those with a nocturnal sleep type (Zhu et al., 2023). Internet leisure negatively affects the amount and quality of sleep through bedtime procrastination (Meng and Anise, 2020), and smartphone addiction is significantly and positively associated with bedtime procrastination (Liu et al., 2021). Therefore, prolonged swiping short videos before bedtime will likely leads to enhanced sleep procrastination behaviors and decreased sleep quality. Hypothesis 3: Procrastination behavior mediates the relationship between short video addiction and sleep quality.

### The chain intermediary role of physical activity and procrastination behavior

There is an essential link between physical activity and procrastination behavior. Generally, procrastination behavior is influenced by environmental and personal factors, while physical activity and short video addiction are behavioral factors. It has been found that procrastination is negatively related to physical activity (Tao et al., 2022). Procrastination affects college students' physical activity through time efficiency (Zhang Y. et al., 2022). Research on academic procrastination has shown that physical activity interventions reduce academic procrastination (Li et al., 2022), and physical activity influences their depressive symptoms through the mediation of academic procrastination (Yang et al., 2023). A survey of 510 general female college students in school found that a yoga activity intervention effectively improved depression and sleep quality levels (Zhang, 2022). Physical activity can affect mood (depression, etc.) and even sleep quality through the mediating effect of procrastination behavior. Therefore, Hypothesis 4: Physical activity and procrastination behavior mediate the relationship between short video addiction and sleep quality.

## Materials and methods

### Participants

This paper distributes electronic questionnaires to students attending a university in Henan Province, China. A total of 360 questionnaires were administered. The number of valid questionnaires obtained after eliminating invalid questionnaires was 337, thus yielding an effective recovery rate of 93.61%. The ages of the survey respondents ranged from 17 to 25 years old (M ± SD = 20.93 ± 1.68 years). There were 170 (50.4%) male students and 167 (49.6%) female students. A total of 62 (18.4%) students were freshman, 73 students (21.66%) were sophomore, 126 (37.39%) students were junior, and 76 (22.55%) students were senior. A total of 187 (55.5%) were physical education majors, and 150 (44.5%) were not physical education majors.

After obtaining consent from the subjects, the surveys were administered online and adhered to the rules of voluntary completion, confidentiality, and anonymity. The gender and grade level of the subjects were controlled. It took ~10–15 min to complete. All data were confidential.

### Measures

#### Short Video Addiction Scale

This study used the Short Video Addiction Scale for College Students, which was developed by Qin (2020). This scale consists of 14 items covering four dimensions: withdrawal (five items), avoidance (three items), loss of control (four items), and inefficacy (two items). The internal consistency reliability coefficient is 0.91, and the Cronbach alpha coefficient of each dimension is also between 0.76 and 0.89. The Cronbach's alpha in this study was 0.94.

#### Pittsburgh Sleep Quality Index

The Pittsburgh Sleep Quality Index (PSQI) was developed and revised by Buysse et al. (1989). It is used to assess the subject's sleep quality in the last month, with higher scores indicating poorer sleep quality. The scale comprises a total of 18 elements for self-assessment. Each item is summed to calculate the total score of the PSQI, and each individual item is evaluated using a scoring system that spans from 0 to 3 points. The Cronbach's alpha coefficient in this investigation was determined to be 0.80.

#### Physical Activity Rating Scale

The measurement of physical activity was conducted by the utilization of the Physical Activity Rating Scale (PARS-3), which was revised by Liang (1994). This scale assesses the amount of exercise across 3 aspects of physical activity: intensity, time, and frequency. Each aspect is scored on a 5 point scale (1–5 points). The Cronbach's alpha in this study was 0.76.

#### Aitken Procrastination Inventory

The Aitken Procrastination Inventory (API) is a 19-question self-report scale developed in 1982 to assess procrastination behavior (Aitken, 1982). A five-point scale is used for each item, and higher scores indicate more severe procrastination behavior. The internal consistency reliability coefficient was 0.82. In this investigation, the Cronbach's alpha was 0.94.

### Statistical analyses

In the present investigation, Pearson correlation and Descriptive analyses of the total scores of the scale and its dimensions were conducted in this study using SPSS 27.0 and its plug-ins. Standard method bias of the data was examined using Harman's one-way test, yielding findings indicating that there were 20 factors with eigenvalues >1. The variation explained by the first factor was 21.29%, well-below critical value of 40%, indicating no standard severe method bias in this study.

## Results

### Descriptive statistics and correlation analysis of the research variables

The mean scores on the Short Video Addiction scale, PSQI, PARS-3, and API were 33.98 ± 13.53, 5.72 ± 3.14, 24.31 ± 24.24, and 51.6 ± 8.759, respectively. Based on the cutoff value for the PSQI of ≥8 (Liu et al., 1994), 25.2% (85 survey respondents) of college students in this survey have poor sleep quality. The correlations among all variables are shown in Table 1. Pearson correlation analysis showed that short video addiction score was positively correlated with sleep quality and procrastination behavior score and negatively correlated with physical activity score. The sleep quality score was negatively correlated with the physical activity score and positively correlated with the procrastination behavior score.

**Table 1:** The main variables and their correlation analysis.

	**M**	**SD**	**Short video addiction**	**Sleep quality**	**Physical activity**	**Procrastination behavior**
Short video addiction	33.98	13.53	1	–	–	–
Sleep quality	5.72	3.14	0.456^**^	1	–	–
Physical activity	24.31	24.24	−0.193^**^	−0.262^**^	1	–
Procrastination behavior	51.6	8.759	0.278^**^	0.273^**^	−0.201^**^	1

*N* = 337.

^**^P < 0.01.

### Analysis of the mediating effect

The data were analyzed using SPSS 27.0 and Hayes' (2013) process macro. The utilization of Model 6 within the process macro was employed to test the mediating effects of physical activity and procrastination behavior on the relationship between short video addiction and sleep quality, using gender, age, and grade as control variables. The results (Table 2) showed that short video addiction positively predicted sleep quality (β = 0.458, *P* < 0.001), significantly and positively predicted procrastination behavior (β = 0.246, *P* < 0.001), and significantly negatively predicted physical activity (β = −0.183, *P* < 0.001). When both physical activity and procrastination behavior were entered into the regression equation, physical activity significantly negatively predicted sleep quality (β = −0.184, *P* < 0.001), procrastination behavior significantly positively predicted sleep quality (β = 0.125, *P* < 0.05), and short-video addiction (β = 0.389, *P* < 0.001) remained significantly positive.

**Table 2:** Regression analysis of variable relationships.

**Regression equation**		**Overall fit index**			**Significance of regression coefficient**		
**Result variable**	**Predictive variable**	***R***	**R2**	***F***	β	**SE**	***t***
Sleep quality	Gender	0.458	0.210	17.545	−0.039	0.324	−0.762
	Age				0.004	0.158	0.043
	Grade				−0.017	0.258	−0.200
	Short-video addiction				0.458	0.012	9.264^***^
Physical activity	Gender	0.378	0.143	11.044	−0.185	2.605	−3.435^***^
	Age				0.092	1.267	1.052
	Grade				0.141	2.077	1.603
	Short-video addiction				−0.183	0.092	−3.551^***^
Procrastination behavior	Gender	0.360	0.130	8.195	−0.098	0.967	−1.773
	Age				0.035	0.463	0.395
	Grade				0.052	0.760	0.586
	Short-video addiction				0.246	0.034	4.655^***^
	Physical activity				−0.205	0.020	−3.691^***^
Sleep quality	Gender	0.511	0.261	16.562	−0.066	0.321	−1.285
	Age				0.019	0.153	0.227
	Grade				0.006	0.252	0.075
	Short-video addiction				0.389	0.012	7.712^***^
	Physical activity				−0.184	0.007	−3.519^***^
	Procrastination behavior				0.125	0.018	2.450^*^

^*^P < 0.05; ^***^P < 0.001.

**Figure 1:**
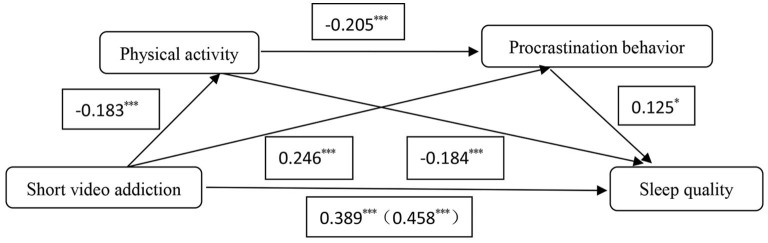
Mediation model of physical activity and procrastination behavior between short video addiction and sleep quality. *P < 0.05; ***P < 0.001.

The results derived from the mediation analysis (see Table 3; Figure 1) showed that physical activity and procrastination behavior significantly mediated the relationship between short video addiction and sleep quality. The total mediating effect value is 0.016, which accounts for 15.07% of the overall effect of short video addiction on sleep quality (effect value 0.106). The mediating effects especially included three pathways' indirect impacts: indirect effect 1 from short video addiction → physical activity → sleep quality (effect value 0.008); indirect effect 2 from short video addiction → procrastination behavior → sleep quality pathway (effect value 0.007); and indirect effect 3 (effect value 0.001) from the short video addiction → physical activity → procrastination behavior → sleep quality pathway. Indirect effects 1, 2 and 3 accounted for 7.34, 6.69, and 1.04% of the total effect, respectively. The 95% confidence intervals for the above indirect effects did not include 0, indicating that all three indirect effects were significant.

**Table 3:** Test of chain-mediated model effect.

**Benefit type**	**Effect value**	**BootSE**	**Bootstrap 95% CI**		**Proportion of relative effect**
			**Boot LLCI**	**Boot ULCI**	
Total effect	0.106	0.012	0.084	0.129	
Direct effect	0.090	0.012	0.067	0.113	84.93%
Indirect effect1	0.008	0.003	0.002	0.014	7.34%
Indirect effect2	0.007	0.003	0.001	0.014	6.69%
Indirect effect3	0.001	0.001	0.001	0.003	1.04%
Total indirect effect	0.016	0.005	0.007	0.026	15.07%

## Discussion

This research investigated the association between short video addiction and sleep quality among college students and examined the underlying mechanism. The findings suggest that short video addiction can affect sleep quality through the chain-mediated effects of physical activity and procrastination behavior. This provides theoretical support for improving sleep quality.

### The influence mechanism of short video addiction on sleep quality

This study discovered a substantial predictive relationship between short video addiction and sleep quality. The higher the degree of short-video addiction among college students, the higher the sleep quality score and the worse the sleep quality. According to multiple earlier studies, short video addiction is a factor that significantly affects college students' ability to sleep (Cain and Gradisar, 2010; Hu et al., 2021). College students often use their cell phones to watch short videos before going to bed (Kroese et al., 2014), and the ease of use and entertainment features of short video apps reinforce the tendency of college students to overuse them, making it difficult to inhibit impulses and potentially increasing emotional exhaustion. The contemplation and emotional engagement triggered by smartphone use at night can cause users to lose sleep (Woods and Scott, 2016; Liu et al., 2017b), while electromagnetic radiation from electronic devices such as cell phones and LED lights suppresses melatonin and may further exacerbate bedtime delays (Andrew et al., 2006; Lemola et al., 2015). Therefore, short video addiction can lead to poorer sleep quality.

### Mediating effect of physical activity

This study found that short video addiction can affect sleep quality through the separate mediation of physical activity. Physical activity can reduce short video addiction and improve sleep quality. Previous research have noted that physical activity positively affects sleep quality, particularly sleep depth, latency, and performance (Anita et al., 2016; Kristine et al., 2018). Related research in China have also found that physical activity significantly directly predicts adolescent sleep quality (Liu et al., 2017a). Engaging in active leisure activities with high-intensity and low-intensity activity results in significant benefits related to the quality of sleep (Passos et al., 2010; Chen et al., 2015). Increased physical activity, especially leisure-related activity, leads to th secretion of endorphins from the pituitary gland, thereby inhibiting addiction by competing for receptors with addictive substances in the central nervous system and inducing feelings of wellbeing (Jovic and Dindic, 2011). Therefore, when college students exhibit high levels of physical activity, they can inhibit the development of addictive behaviors and improve the sleep quality.

### Mediating effect of procrastination behavior

Short video addiction can affect sleep quality via procrastination behavior. Negative emotions (e.g., anxiety) induced by mobile phone addiction are essential triggers of procrastination behavior (Jun, 2016); therefore, short video addiction among college students increases sleep procrastination, which in turn lowers the quality of their sleep (Dong, 2022). One of the most important consequences of media use on sleep is that it causes sleep to be both postponed and abbreviated (Van den Bulck, 2010). As more time is devoted to media, less time is devoted to other activities, including sleep. Short nighttime videos provide the most convenient medium for individuals' procrastination behavior, which leads to increasing procrastination and the negative emotions associated with individuals' procrastination behaviors, which directly affects their attitudes and behaviors and brings about sleep delays. Studies have shown that TikTok addicts are at a more significant disadvantage in terms of mental health (Chao et al., 2023); therefore, the more severe the addiction to short videos, the more pronounced the procrastination behaviors and the poorer quality of sleep will be.

### The chain intermediary roles of college students' physical activity and procrastination behavior

This investigation discovered two independent mediators, i.e., physical activity and procrastination behavior, further affected sleep quality as follows: short video addiction → physical activity → procrastination behavior → sleep quality. This result suggests that college students' short-video addiction influences sleep quality via the chain mediating effects of physical activity and procrastination behavior. Regression analyses controlling for age, gender, and grade level revealed that short video addiction significantly predicted sleep quality, and the greater the extent of college students' addiction to short videos, the greater the likelihood that they would develop sleep disorders, which agrees with earlier research (Sebastian and Wang, 2021). The specific reason for this may be that immersive behaviors are enormously appealing to individuals, thus enabling people to engage in them for long periods (Jiao et al., 2023). Short videos generally have the effect of immersing people for a short period, and often, a video of 10 s can make people unconsciously engage in it. College students are the most sensitive group to the outside world's fresh information and active thinking. Short video applications easily influence them, so it is easy to impact the inherent sleep rhythm in the case of short video addiction. A survey of 839 college students found that “watching videos and playing games” was the main factor in college students' sleep delay, accounting for 21.81% (Li et al., 2023). College students with serious short video addiction are prone to neglect the surrounding environment and the completion of study tasks and participate in fewer physical activities, which is more likely to result in delayed behavior, sleep disorders, and poorer quality sleep.

### Limitations and future directions

Although the study's hypotheses were validated, its research still has limitations. First, the study was cross-sectional, in which all information was collected at once and lacked a longitudinal follow-up study. Second, the design of the questionnaire needs to be improved, with insufficient consideration of other external variables; the questionnaire needs to be filled out in a standardized way, which biases the objectivity of the data. Therefore, further research should consider adding a longitudinal design or experimental intervention to the study, which could help establish causal relationships. Consider adding factors such as nationality, age groups, and other external variables such as Body Mass Index (BMI) and sedentary time. Enhance the accuracy and reliability of measurements by using more objective measurement devices for physical activity levels, sleep quality, and short video addiction. Deepen the exploration of relationships within related variables, such as the impact of increased and decreased physical activity on procrastination behavior. In future studies, we can expand the investigation of the long-term effects of video addiction on sleep quality and the role of physical activity and procrastination, explore interventions targeting physical activity or procrastination to mitigate the adverse effects of video addiction on sleep quality, and help college students improve their sleep quality more effectively.

## Conclusion

The results of study indicate that short video addiction significantly predicts sleep quality among college students and that physical activity and procrastination behavior significantly mediate the relationship between short video addiction and sleep quality. This study further affirms the role and significance of physical activity in decreasing short video addiction and procrastination behavior and improving sleep quality. The study's results can help deepen college students' understanding of short video addiction, help them recognize the importance of physical activity, and assist in effectively improving their sleep quality. The study suggests that colleges and universities should consider the following recommendations: improve the culture of university athletics; foster a positive sports environment for college students; civilize their spirit and salvage their bodies; promote mass sports on campus; hold campus fitness runs; plan friendly basketball matches, calisthenics competitions and other activities; and provide more time and chances for college students to participate in healthy, physically and mentally nourishing sports. These activities can lead to improvements in the procrastination behaviors of college students, thereby effectively alleviating short video addiction and improving sleep quality.

## Data availability statement

The original contributions presented in the study are included in the article/supplementary material, further inquiries can be directed to the corresponding author.

## Ethics statement

Ethical review and approval was not required for the study on human participants in accordance with the local legislation and institutional requirements. Written informed consent from the patients/participants or patients/participants legal guardian/next of kin was not required to participate in this study in accordance with the national legislation and the institutional requirements.

## Author contributions

ZZ: Writing – original draft, Writin – review & editing. YK: Investigation, Writing – review & editing.

## References

[B1] Aili K. Åström-Paulsson S. Stoetzer U. Svartengren M. Hillert L. (2017). Reliability of actigraphy and subjective sleep measurements in adults: the design of sleep assessments. J. Clin. Sleep Med. 13, 39–47. 10.5664/jcsm.6384 27707448 PMC5181612

[B2] Aitken M. E. (1982). A Personality Profile of the College Student Procrastinator. Pittsburgh, PA: University of Pittsburgh.

[B3] Andrew W. Wood S. Loughran P. Stough C. (2006). Does evening exposure to mobile phone radiation affect subsequent melatonin production? Int. J. Radiat. Biol. 82, 69–76. 10.1080/09553000600599775 16546905

[B4] Aneta P. Błachnio A. Yat-Fan Siu N. (2019). The relationships between self-efficacy, self-control, chronotype, procrastination and sleep problems in young adults. Chronobiol. Int. 36, 1025–1035. 10.1080/07420528.2019.1607370 31070062

[B5] Anita D. C. Seery E. Kent J. A. (2016). Physical activity, sleep quality, and self-reported fatigue across the adult lifespan. Exp. Gerontol. 77, 7–11. 10.1016/j.exger.2016.02.001 26853493 PMC4808431

[B6] Buysse D. J. Reynolds C. F. III. Monk T. H. Kupfer D. J. (1989). The Pittsburgh sleep quality index: a new instrument for psychiatric practice and research. Psychiatry Res. 28, 193–213 10.1016/0165-1781(89)90047-4 2748771

[B7] Cain N. Gradisar M. (2010). Electronic media use and sleep in school-aged children and adolescents: a review. Sleep Med. 11, 735–742. 10.1016/j.sleep.2010.02.006 20673649

[B8] Chao M. Lei J. He R. Jiang Y. Yang H. (2023). TikTok use and psychosocial factors among adolescents: Comparisons of non-users, moderate users, and addictive users. Psychiatry Res. 325, 115247. 10.1016/j.psychres.2023.115247 37167877

[B9] Chen L.-J. Fox K. R. Ku P.-W. Chang Y.-W. (2015). Effects of aquatic exercise on sleep in older adults with mild sleep impairment: a randomized controlled trial. Int. J. Behav. Med. 23, 501–506. 10.1007/s12529-015-9492-0 26025630

[B10] China Internet Network Information Center (2023). The 51st Statistical Report on the Development of the Internet in China. Available online at :https://cnnic.cn/n4/2023/0302/c199-10755.~html (accessed August 10, 2023).

[B11] Dissing A. S. Andersen T. O. Nørup L. N. Clark A. Nejsum M. Rod N. H. (2022). Daytime and nighttime smartphone use: A study of associations between multidimensional smartphone behaviours and sleep among 24, 856 Danish adults. J. Sleep Res. 30, 313356. 10.1111/jsr.13356 33899250

[B12] Dong W. (2022). The Effects of Short Video Addiction on Sleep Quality Among College Students: The Chain-Mediated Roles of Self-Regulation and Sleep Procrastination. Changchun: Jilin University, 6–7.

[B13] Fuschia M. S. van Eerde E. Argiropoulou I. (2015). Is procrastination related to sleep quality? Testing an application of the procrastination–health model. Cogent. Psychol. 2, 1. 10.1080/23311908.2015.1074776

[B14] Ghrouz A. K. Noohu M. M. Dilshad Manzar M. Warren Spence D. BaHammam A. S. Pandi-Perumal S. R. (2019). Physical activity and sleep quality in relation to mental health among college students. Sleep Breath. 23, 627–634. 10.1007/s11325-019-01780-z 30685851

[B15] Guo M. J. Li N. Zhao Y. Wang P. You S. M. Wang Q.. (2023). Mediating effect of loneliness between short video addiction and sleep quality among undergraduate nursing students. J. Nurs. 30, 20–24. 10.16460/j.issn1008-9969.2023.07.020

[B16] Hayes A. (2013). Introduction to mediation, moderation, and conditional process analysis. J. Educ. Meas. 51, 335–337. 10.1111/jedm.12050

[B17] Hu W. Jiang Y. Wang Q. Wang N. (2021). The relationship between short-video social media dependence and sleep disorders in college students: the mediating role of nighttime social media use and gender differences. Chin. J. Clin. Psychol. 29, 46–50. 10.16128/j.cnki.1005-3611.2021.01.009

[B18] Jiao Y. Ma Y. Li Y. F. (2023). Analysis of sleep procrastination behavior and psychological intervention among college students in the information age. J. Jinzhou Med. Univ. 21, 60–63.

[B19] Jovic J. Dindic N. (2011). Influence of dopaminergic system on internet addiction. Acta Med. Median 50, 60–66. 10.5633/amm.2011.0112

[B20] Jun S. (2016). The reciprocal longitudinal relationships between mobile phone addiction and depressive symptoms among Korean adolescents. Comp. Hum. Behav. 58, 179–186. 10.1016/j.chb.2015.12.061

[B21] Katz E. Blumler J. G. (1976). The uses of mass communication: current perspectives on gratifications research. Am. J. Sociol. 81, 1546–1548. 10.1086/226259

[B22] Kristine A. W. Erickson K. I. Wheeler M. E. (2018). Physical activity and cognition: a mediating role of efficient sleep. Behav. Sleep Med. 16, 569–586. 10.1080/15402002.2016.1253013 27935322 PMC5466488

[B23] Kroese F. M. De Ridder D. T. D. Evers C. Adriaanse M. A. (2014). Bedtime procrastination: introducing a new area of procrastination. Front. Psychol. 5,611. 10.3389/fpsyg.2014.00611 24994989 PMC4062817

[B24] Lemola S. Perkinson-Gloor N. Brand S.. (2015). Adolescents' electronic media use at night, sleep disturbance, and depressive symptoms in the smartphone age. J. Youth Adolesc. 44, 405–418. 10.1007/s10964-014-0176-x 25204836

[B25] Li C. Hu Y. Ren K. (2022). Physical activity and academic procrastination among Chinese university students: a parallel mediation model of self-control and self-efficacy. Int. J. Environ. Res. Public Health 19, 6017. 10.3390/ijerph19106017 35627552 PMC9140729

[B26] Li J. Y. Luan J. Zhang W. Y. Li H. J. (2023). Exploration of the status quo and influencing factors of sleep procrastination behavior of 839 college students in a city. Chin. Sch. Nurse 37, 241–254.

[B27] Liang D. Q. (1994). Stress level of college students and its relationship with physical exercise. Chin. J. Mental Health 1994, 5–6.

[B28] Liu H. Ji Y. Dust S. B. (2021). “Fully recharged” evenings? The effect of evening cyber leisure on next-day vitality and performance through sleep quantity and quality, bedtime procrastination, and psychological detachment, and the moderating role of mindfulness. J. Appl. Psychol. 106, 990–1006. 10.1037/apl0000818 32816502

[B29] Liu Q. Q. Zhou Z. K. Niu G. F. Fan C. Y. (2017a). Mobile phone addiction and sleep quality in adolescents: mediation and moderation analyses. Acta Psychol. Sinica. 49, 1524–1536. 10.3724/SP.J.1041.2017.01524

[B30] Liu Q. Q. Zhou Z. K. Yang X. J. Kong F. C. Niu G. F. Fan C. Y. (2017b). Mobile phone addiction and sleep quality among Chinese adolescents: a moderated mediation model. Comput. Hum. Behav. 72, 108–114. 10.1016/j.chb.2017.02.04232751334

[B31] Liu X. C. Tang M. Q. Hu L. Wang A. Z. Chen K. Zhao G. F. (1994). Correlation between sleep quality and mental health status of college students. Shandong Psychiatry. 4, 4–9.

[B32] Ma X. Meng D. Zhu L. Xu H. Guo J. Yang L.. (2022). Bedtime procrastination predicts the prevalence and severity of poor sleep quality of Chinese undergraduate students. J. Am. Coll. Health 70, 1104–1111. 10.1080/07448481.2020.1785474 32669056

[B33] Mahfouz M. S. Ali S. A. Bahari A. Y. Ajeebi R. E. Sabei H. J. Somaily S. Y.. (2020). Association between sleep quality and physical activity in Saudi Arabian University Students. Nat. Sci. Sleep 12, 775–782 10.2147/NSS.S267996 33117013 PMC7585794

[B34] Mega Arithmetic (2022). Jitterbug Youth Observation Report. Available online at: https://trendinsight.oceanengine.~com/arithmetic-report/detail/749 (accessed August 10, 2023).

[B35] Meng X. Z. Anise M. S. (2020). Effects of smartphone addiction on sleep quality among Chinese university students: The mediating role of self-regulation and bedtime procrastination. Addict. Behav. 111, 106552. 10.1016/j.addbeh.2020.106552 32717501

[B36] Passos G. S. Poyares D. Santana M. G. Garbuio S. A. Tufik S. Mello M. T. (2010). Effect of acute physical exercise on patients with chronic primary insomnia. J. Clin. Sleep Med. 6, 270–275. 10.5664/jcsm.27825 20572421 PMC2883039

[B37] Pereira F. S. Bevilacqua G. G. Coimbra D. R. Andrade A. (2020). Impact of problematic smartphone use on mental health of adolescent students: association with mood, symptoms of depression, and physical activity. Cyberpsych 23, 619–626. 10.1089/cyber.2019.0257 32580574

[B38] Pu H. Leong R. F. L. Michael W. L. (2022). Bedtime procrastination and chronotype differentially predict adolescent sleep on school nights and non-school nights. Sleep Health 8, 640–647. 10.1016/j.sleh.2022.09.007 36272919

[B39] Pu Y. X. Yang D. H. Yan W. Y. (2023). The relationship between negative life events and short video addiction among college students: the mediating role of self-compensatory motivation. J. Nanjing Univ. Trad. Chin. Med. 24, 204–210.

[B40] Qin H. X. (2020). Research on the influence mechanism and intervention countermeasures of short video addiction among college students. Jiangxi Norm. Univ. Sci. Technol. 15-17. 10.27751/d.cnki.gjxkj.2020.000299

[B41] Qin H. X. Li X. Zeng M. H. He Y. X. (2019). Preliminary development of short video addiction scale for college students. Front. Chin. Psychol. 1, 586–598. 10.35534/pc.0108037

[B42] Rathakrishnan B. Singh S. S. B. Kamaluddin M. R. Yahaya A. Nasir M. A. M. Ibrahim F.. (2021). Smartphone addiction and sleep quality on academic performance of university students: an exploratory research. Int. J. Environ. Res. Public Health. 18, 8291. 10.3390/ijerph18168291 34444042 PMC8394437

[B43] Sebastian S. Wang K. (2021). Explaining the success of social media with gratification niches: Motivations behind daytime, nighttime, and active use of TikTok in China. Comp. Hum. Behav. 124, 106893. 10.1016/j.chb.2021.106893

[B44] Semplonius T. Willoughby T. (2018). Long-term links between physical activity and sleep quality. Med. Sci. Sports Exerc. 50, 2418–2424. 10.1249/MSS.0000000000001706 30048409

[B45] Tao Y. Yu H. Liu M. Wang P. Zhang J. Yang Y.. (2022). Procrastination and physical activity: the moderated mediating effect of grit. J. Am. Coll. Health 2022, 1–9. 10.1080/07448481.2022.2068962 35549652

[B46] Tian H. J. (2019). The effects of high perfectionist standards on college students' procrastination behavior: a chain-mediated effect analysis. Psychol. Behav. Res. 17, 668–674.

[B47] Tian X. Bi X. Chen H. (2023). How short-form video features influence addiction behavior? Empirical research from the opponent process theory perspective. Inf. Technol. People 36, 387–408. 10.1108/ITP-04-2020-0186

[B48] Van den Bulck J. (2010). The effects of media on sleep. Adolesc. Med. State Art Rev. 21, 418–429. 10.1542/9781581105803-the_effects 21302852

[B49] Wang F. Boros S. (2021). The effect of physical activity on sleep quality: a systematic review. Eur. J. Physiother. 23, 11–18. 10.1080/21679169.2019.1623314

[B50] Wang K. Scherr S. (2022). Dance the night away: how automatic tiktok use creates pre-sleep cognitive arousal and daytime fatigue. Mobile Media Commun. 10, 316–336. 10.1177/20501579211056116

[B51] Woods H. C. Scott H. (2016). Sleepyteens: social media use in adolescence is associated with poor sleep quality, anxiety, depression and low self-esteem. J. Adolesc. 51, 41–49. 10.1016/j.adolescence.2016.05.008 27294324

[B52] Xie Y. Wu X. Y. Tao S. M. Xiang J. M. Xu Y. S. Yang Y. J.. (2020). The relationship between cell phone dependence and anxiety and sleep quality among college students. China Sch. Health 11, 1621–1624. 10.16835/j.cnki.1000-9817.2020.11.006

[B53] Yang L. Liu Z. Shi S. Dong Y. Cheng H. Li T. (2023). The mediating role of perceived stress and academic procrastination between physical activity and depressive symptoms among Chinese college students during the COVID-19 pandemic. Int. J. Environ. Res. Public Health. 20, 773. 10.3390/ijerph20010773 36613096 PMC9819869

[B54] Zhai X. Wu N. Koriyama S. Wang C. Shi M. Huang T.. (2021). Mediating effect of perceived stress on the association between physical activity and sleep quality among Chinese college students. Int. J. Environ. Res. Public Health. 18, 289. 10.3390/ijerph18010289 33401720 PMC7795525

[B55] Zhang J. (2022). Effects of Yoga Exercise Intervention on Depression and Sleep Quality in Female College Students. Shanghai: Shanghai Institute of Physical Education, 22–23.

[B56] Zhang X. Feng S. Peng R. Li H. (2022). Using structural equation modeling to examine pathways between physical activity and sleep quality among chinese tiktok users. Int. J. Environ. Res. Public Health. 19, 5142. 10.3390/ijerph19095142 35564536 PMC9105446

[B57] Zhang Y. Yang J. Wu M. Yuan Y. (2022). Relationship between procrastination and exercise behavior among college students: the mediating effects of time efficiency. Int. J. Phys. Act. Health 20, 2. 10.18122/ijpah.1.2.20.boisestate

[B58] Zhao H. Lu C. Yi C. (2023). Physical activity and sleep quality association in different populations: a meta-analysis. Int. J. Environ. Res. Public Health. 20, 1864. 10.3390/ijerph20031864 36767229 PMC9914680

[B59] Zhou L. Sui J. L. Wang C. Q. (2019). A study on the relationship between college students' sleep quality and mobile phone addiction. Psychol. Monthly 14, 25–27. 10.19738/j.cnki.psy.2019.18.013

[B60] Zhu Y. Huang J. Yang M. (2023). Association between chronotype and sleep quality among Chinese college students: the role of bedtime procrastination and sleep hygiene awareness. Int. J. Environ. Res. Public Health 20, 197. 10.3390/ijerph20010197 36612519 PMC9820042

